# Short-term outcomes after laparoscopic colorectal cancer surgery in patients over 90 years old: a Japanese multicenter study

**DOI:** 10.1186/s12893-023-02298-8

**Published:** 2024-01-02

**Authors:** Mariko Yamashita, Tetsuro Tominaga, Takashi Nonaka, Makoto Hisanaga, Hiroaki Takeshita, Hidetoshi Fukuoka, Kazuo To, Kenji Tanaka, Terumitsu Sawai, Takeshi Nagayasu

**Affiliations:** 1 Department of Surgical Oncology, Nagasaki University Graduate School of Biomedical Science, 1-7-1 Sakamoto, Nagasaki, 852-8501 Japan; 2 Department of Surgery, Sasebo City General Hospital, Nagasaki, Japan; 3 Department of Surgery, National Hospital Organization Nagasaki Medical Center, Nagasaki, Japan; 4 Department of Surgery, Isahaya General Hospital, Isahaya, Japan; 5 Department of Surgery, Ureshino Medical Center, Ureshino, Japan; 6 Department of Surgery, Saiseikai Nagasaki Hospital, Nagasaki, Japan

**Keywords:** Laparoscopic Surgery, Over 90 years, Postoperative complication

## Abstract

**Background:**

The effect of laparoscopic surgery on short-term outcomes in colorectal cancer patients over 90 years old has remained unclear.

**Methods:**

We reviewed 87 colorectal cancer patients aged over 90 years who underwent surgery between 2016 and 2022. Patients were divided into an open surgery group (n = 22) and a laparoscopic surgery group (n = 65). The aim of this study was to investigate the effect of laparoscopic surgery on postoperative outcome in elderly colorectal cancer patients, as compared to open surgery.

**Results:**

Seventy-eight patients (89.7%) had comorbidities. Frequency of advanced T stage was lower with laparoscopic surgery (*p* = 0.021). Operation time was longer (open surgery 146 min vs. laparoscopic surgery 203 min; *p* = 0.002) and blood loss was less (105 mL vs. 20 mL, respectively; *p* < 0.001) with laparoscopic surgery. Length of hospitalization was longer with open surgery (22 days vs. 18 days, respectively; *p* = 0.007). Frequency of infectious complications was lower with laparoscopic surgery (18.5%) than with open surgery (45.5%; *p* = 0.021). Multivariate analysis revealed open surgery (*p* = 0.026; odds ratio, 3.535; 95% confidence interval, 1.159–10.781) as an independent predictor of postoperative infectious complications.

**Conclusions:**

Laparoscopic colorectal resection for patients over 90 years old is a useful procedure that reduces postoperative infectious complications.

## Background

Opportunities to treat very elderly patients with colorectal cancer are increasing, as colorectal cancer is the most frequent cancer in the elderly [1, 2]. In recent years, the Japanese and global populations have been aging, with 5.2% of the population ≥85 years old and 2.1% ≥90 years old in Japan and 0.86% and 0.29%, respectively, worldwide [3].

On the other hand, many elderly patients have problems with age-related organ dysfunction like cardiovascular or respiratory diseases [4, 5]. Given fears of mortality from operative invasion, elderly patients tend to be treated more conservatively than non-elderly patients.

Laparoscopic colorectal resection (LCR) has become popular in recent years as a minimally invasive approach. Various randomized controlled trials (RCTs) have shown that LCR provides better short-term surgical outcomes than open surgery, but all such RCTs have excluded patients ≥90 years old [6–11]. One retrospective study that examined reported surgical outcomes for colorectal cancer patients ≥90 years old included fewer cases using LCR compared to an open approach.

The aim of this multicenter study was to investigate the effect of LCR on postoperative outcome in elderly colorectal cancer patients, as compared to open surgery.

## Methods

We reviewed data collected from consecutive patients ≥90 years old who underwent surgery for colorectal cancer in Nagasaki University Hospital and five other participating hospitals (Sasebo City General Hospital, Nagasaki Medical Center, Isahaya General Hospital, Ureshino Medical Center, and Saiseikai Nagasaki Hospital) between April 2016 and February 2022.

Informed consent was obtained from these patients for the use of their data in this study. This study was performed in line with the principles of the Declaration of Helsinki. Approval was granted by the clinical research review boards of all participating hospitals.

Patients were divided into two groups: the group that had undergone open colorectal resection (OP group) and the group that had undergone laparoscopic colorectal resection (LCR group). Indications for the surgical procedure were selected by the attending physician based on their personal judgment, taking into account the condition of the patient and the progression of the tumor. Patient data were collected to compare clinical characteristics: sex; age; body mass index (BMI); American Society of Anesthesiologists (ASA) performance status (PS); comorbidities; and clinical T/N/M stage. Tumor location was classified as either in the rectum or in the colon. Surgical and pathological data collected included: the type of procedure; operation time; volume of blood loss; use of open conversion; postoperative complications; and duration of hospitalization. We recorded the single complication that most affected the clinical course and its severity according to the Clavien–Dindo (CD) grade. Patients with CD grade 2 or higher complications were assigned to the complication group. Postoperative complications were defined as those occurring within 30 days postoperatively.

Data are presented as median values with ranges. Differences in categorical variables were compared using Fisher’s exact test or the chi-squared test. Differences in continuous variables were analyzed using the Mann–Whitney U-test. In multivariate analysis, logistic regression analysis was used to identify risk factors for postoperative complications. Cut-off values for operative time and blood loss were established by receiver operating characteristic analysis. Variables with a value of *P* < 0.05 in univariate analyses were included in the multivariate analysis. All values of *P* < 0.05 were considered significant. Statistical analysis was performed using Bell Curve for Excel software (version 2.02; Social Survey Research Information Co., Tokyo, Japan).

## Results

Patients who underwent emergency surgery or who had synchronous colon cancer or incomplete laboratory data or only bypass or stoma were excluded. A final total of 87 patients were included for analysis, with 22 patients in the OP group and 65 patients in the LCR group.

Table 1 shows the clinical and surgical features of the 87 patients (53 men, 34 women; median age, 92 years; range, 90–98 years). Median BMI was 19.9 kg/m^2^ (range, 12.9–30.0 kg/m^2^). Comorbidities were present in 78 patients (89.7%) and no patients had received preoperative treatment. Most patients had ascending colon cancer or rectal cancer (n = 21 each, 24.1%). Median operation time was 187 min (range, 60–560 min) and median blood loss was 35 mL (range, 0–843 mL). In 1 case (1.1%), the laparoscopic approach was converted to open surgery. Postoperative complications (CD ≥2) occurred in 35 patients (40.2%). Median hospital stay was 19 days (range, 7–140 days).

**Table 1:** Patient characteristics

	All patients (*n* = 87) (%)
Sex	
Male	34 (39.1)
Female	53 (60.9)
Age, y (range)	92 (90–98)
Body mass index, kg/m^2^ (range)	19.9 (12.9–30.0)
ASA performance status	
1	9 (10.3)
2	50 (57.5)
3	27 (31.0)
4	1 (1.1)
Comorbidities, present	78 (89.7)
Hypertension	44 (50.5)
Diabetes mellitus	14 (16.1)
Heart failure	13 (14.9)
Dementia	9 (10.3)
Angina pectoris / myocardial infarction	7 (8.0)
Valvular disease	7 (8.0)
Other cancer	6 (6.9)
Renal failure	6 (6.9)
Arrhythmia	6 (6.9)
Chronic obstructive pulmonary disease	4 (4.6)
Collagen disease	1 (1.1)
Tumor location	
Cecum	12 (13.8)
Ascending colon	21 (24.1)
Transverse colon	15 (17.2)
Descending colon	5 (5.7)
Sigmoid colon	13 (14.9)
Rectum	21 (24.1)
Operative procedure	
Ileocecal resection	20 (23.0)
Hemicolectomy	21 (24.1)
Partial resection	9 (10.3)
Sigmoidectomy	9 (10.3)
Anterior resection	9 (10.3)
Hartmann	10 (11.5)
Abdominoperineal resection	9 (10.3)
Clinical T factor	
1	7 (8.0)
2	12 (13.8)
3	45 (51.7)
4	23 (26.4)
Clinical N factor	
0	47 (54.0)
1	27 (31.0)
2	12 (13.8)
Distant metastasis, present	7 (8.0)
Operation time, min (range)	187 (60–560)
Blood loss, mL (range)	35 (0–843)
Blood transfusion, yes	22 (25.3)
Open conversion	1 (1.1)
Postoperative complications, CD ≥ 2	35 (40.2)
Duration of urethral catheter removal, days (range)	3 (2–7)
Hospital stay, days (range)	19 (7–140)
Re-admission within 30 days, yes	4 (4.6)

Data are presented as the number of patients (%) or the median (range)

ASA, American Society of Anesthesiologists; CD, Clavien–Dindo

Table 2 shows clinical and surgical differences between the OP and LCR groups. Patients with advanced T stage were less frequent in the LCR group (*p* = 0.021). The LCR group showed a longer operation time (OP 146 min vs. LCR 203 min; *p* = 0.002) and lower volume of blood loss (OP 105 mL vs. LCR 20 mL; *p* < 0.001). Duration of hospitalization was longer in the OP group (OP 22 days vs. LCR 18 days; *p* = 0.007). Other factors including sex, age, BMI, ASA-PS, comorbidities, tumor location, operative procedure, clinical N/M factor, and postoperative complications were similar between groups.

**Table 2:** Comparison of clinical characteristics between the OP group and LCR group

	OP (*n* = 22) (%)	LCR (*n* = 65) (%)	*P*-value
Sex			0.805
Male	8 (36.4)	26 (40.0)	
Female	14 (63.6)	39 (60.0)	
Age, y (range)	92 (90–98)	92 (90–98)	0.418
Body mass index, kg/m^2^	19.7 (12.9–28.8)	20.3 (14.1 − 30.0)	0.281
ASA performance status			0.269
1	3 (13.6)	6 (9.2)	
2	13 (59.1)	37 (56.9)	
3	5 (22.7)	22 (33.8)	
4	1 (4.5)		
Comorbidities			1.000
No	3 (13.6)	6 (9.2)	
Yes	19 (86.4)	59 (90.8)	
Tumor location			1.000
Colon	17 (77.3)	49 (75.4)	
Rectum	5 (22.7)	16 (24.6)	
Operative procedure			0.326
Ileocecal resection	7 (31.8)	13 (20.0)	
Hemicolectomy	5 (22.7)	16 (24.6)	
Partial resection	4 (18.2)	5 (7.7)	
Sigmoidectomy	0 (0)	9 (13.8)	
Anterior resection	1 (4.5)	8 (12.3)	
Hartmann	3 (13.6)	7 (10.8)	
Abdominoperineal resection	2 (9.1)	7 (10.8)	
Clinical T factor			0.021
1	0 (0)	7 (10.8)	
2	2 (9.1)	10 (15.4)	
3	9 (40.9)	36 (55.4)	
4	11 (50.0)	12 (18.5)	
Clinical N factor			0.969
0	11 (50.0)	36 (55.4)	
1	7 (31.8)	20 (30.8)	
2	3 (13.6)	9 (13.8)	
Distant metastasis			0.834
No	20 (90.9)	60 (92.3)	
Yes	2 (9.1)	5 (7.7)	
Operation time, min (range)	146 (60–284)	203 (77–560)	0.002
Blood loss, mL (range)	105 (5–843)	20 (0–641)	0.001
Blood transfusion, yes	7 (31.8)	9 (13.8)	0.107
Open conversion	-	1 (1.5)	
Postoperative complications, CD ≥2	11 (50.0)	24 (36.9)	0.320
Duration of urethral catheter removal, days (range)	4 (3–7)	3 (2–7)	0.052
Hospital stay, days (range)	22 (11–59)	18 (7–107)	0.007
Re-admission within 30-days, yes	2 (9.1)	2 (3.1)	0.264

Data are presented as the number of patients (%) or median (range)

OP, open surgery; LCR, laparoscopic colorectal resection; ASA, American Society of Anesthesiologists

Differences in categorical variables were compared using Fisher’s exact test or the chi-squared test, as appropriate. Differences in continuous variables were analyzed with the Mann–Whitney *U*-test

Table 3 shows the details of postoperative complications. The overall complication rate was similar between groups (OP 50.0% vs. LCR 36.9%; *p* = 0.320). Infectious complications were less frequent in the LCR group (OP 45.5% vs. LCR 18.5%; *p* = 0.021).

**Table 3:** Details of postoperative complications (CD ≥2)

	All cases (n = 87) (%)	OP (*n* = 22) (%)	LCR (*n* = 65) (%)	*P*-value
Postoperative complications	35 (40.2)	11 (50.0)	24 (36.9)	0.320
Infectious complications	22 (25.3)	10 (45.5)	12 (18.5)	0.021
Urinary tract infection	6 (6.9)	3 (13.6)	3 (4.6)	
Pneumonia	6 (6.9)	4 (18.1)	2 (3.1)	
Surgical site infection	5 (5.7)	2 (9.1)	3 (4.6)	
Anastomotic leakage	2 (2.3)	1 (4.5)	1 (1.5)	
Pseudomembranous colitis	2 (2.3)	0 (0)	2 (3.1)	
MRSA colitis	1 (1.1)	0 (0)	1 (1.5)	
Non-infectious complications	13 (14.9)	1 (4.5)	12 (18.5)	0.170
Ileus	9 (10.3)	0 (0)	9 (13.8)	
Arrhythmia	1 (1.1)	0 (0)	1 (1.5)	
Delirium	1 (1.1)	0 (0)	1 (1.5)	
Pneumothorax	1 (1.1)	0 (0)	1 (1.5)	
Stomal prolapse	1 (1.1)	1 (1.1)	0 (0)	

Differences in categorical variables were compared using Fisher’s exact test or the chi-squared test, as appropriate. CD, Clavien–Dindo; OP, open surgery; LCR, laparoscopic colorectal resection; MRSA, methicillin-resistant *Staphylococcus aureus*

Table 4 shows the predictive ability of clinical factors for both postoperative overall complications and postoperative infectious complications. Univariate analysis revealed that sex and blood loss were significantly associated with postoperative overall complications. However, multivariate analysis revealed no independent predictive factors for postoperative overall complications.

**Table 4:** Clinical factors predicting postoperative overall complications and infectious complications among colorectal cancer patients

	Postoperative overall complication				Infectious complication			
	Univariate analysis	Multivariate analysis			Univariate analysis	Multivariate analysis		
	*P* value	Odds ratio	95%CI	*P* value	*P* value	Odds ratio	95%CI	*P* value
Sex	0.045			0.106	0.227			
Female		1						
Male		2.113	0.851–5.243					
ASA performance status	0.202				0.966			
1–2								
3–4								
BMI, kg/m^2^	0.365				0.984			
< 25								
≥25								
Location	0.259				0.254			
Colon								
Rectum								
Blood transfusion	0.826				0.757			
No								
Yes								
Surgical approach	0.176				0.005			0.026
LCR						1		
OP						3.535	1.159–10.781	
Clinical T factor	0.900				0.648			
1–3								
4								
Clinical N factor	0.761				0.669			
Absence								
Presence								
Operation time, min	0.901				0.042			0.270
< 160						1		
≥160						0.538	0.179–1.617	
Estimated blood loss, mL	0.049			0.096	0.421			
< 35		1						
≥35		2.141	0.872–5.256					

HR, hazard ratio; CI, confidence interval; BMI, body mass index; ASA, American Society of Anesthesiologists; OP, open surgery; LCR, laparoscopic colorectal resection

Cox proportional hazards modeling was used to identify independent risk factors for postoperative complications

Univariate analysis revealed that surgical approach and operation time were significantly associated with postoperative infectious complications. Multivariate analysis revealed open surgery (*P* = 0.026; OR, 3.535; 95%CI, 1.159–10.781) as an independent predictor of postoperative infectious complications.

## Discussion

In the present study, we examined the effect of LCR on postoperative complications in colorectal cancer patients ≥90 years old. The proportion of comorbidities was extremely high (89.7%). The LCR group showed longer operation time, smaller volume of blood loss, lower frequency of infectious complications, and shorter duration of hospitalization compared to the OP group. Multivariate analysis revealed LCR as an independent negative predictor of infectious complications.

Elderly individuals are more likely to have comorbidities such as cardiovascular, pulmonary, and renal diseases than younger people, and are considered at high risk for postoperative complications [12–14]. Ghanem et al. studied 71 patients over 90 years old with pertrochanteric fracture [12]. Cardiac disease (44.3%), renal disease (37.1%), and psychiatric disease (37.1%) were significantly more frequent among the elderly than among younger patients. Issa et al. studied patients over 90 years old with traumatic dentate fractures and found that they had a greater frequency of comorbidities involving vital organs, including heart failure (40%), diabetes mellitus (27%), peripheral vascular disease (27%), and myocardial infarction (20%) [13]. The present study examined patients ≥90 years old, finding comorbidities in 89.7%. Important organ complications in this cohort included heart disease (heart failure, 14.9%; angina pectoris/myocardial infarction, 8.0%; valvular disease, 8.0%; arrhythmia, 6.9%; hypertension, 50.5%), renal disease (6.9%), diabetes mellitus (16.1%), and chronic obstructive pulmonary disease (4.6%). These frequencies were slightly lower than those reported previously. One possible explanation is that this study did not consider patients who had not undergone surgery. Chen et al. reported that 29 of 100 colorectal cancer patients ≥90 years old opted for non-operative treatment [3]. The present cohort may have been selected from patients for whom critical organ comorbidity status was acceptable for general anesthesia.

Laparoscopic surgery has been more popular in recent years, and various RCTs have examined short- and long-term outcomes [6–9]. RCTs for colon cancer have shown that LCR for colon cancer patients is feasible and safe compared with open surgery [1–6]. Despite the longer operation time, the LCR group in our study demonstrated better short-term outcomes such as lower volume of blood loss, better recovery of bowel function and shorter duration of hospital stay. In Japan, the JCOG 0404 study evaluated overall survival after LCR for clinical stage II/III colon cancer [6]. The conversion rate to open surgery was 5.4%, but the mortality rate was 0%. Operation time was longer for the LCR group (211 min) than for the OC group (159 min; *p* < 0.001), but LCR resulted in a lower volume of blood loss (LCR 30 mL vs. OC 85 mL; *p* < 0.001), shorter time to first flatus (LCR 1.8 days vs. OC 2.4 days; *p* < 0.001), less use of analgesics (LCR 175 times per 5 postoperative days vs. OC 241 times per 5 postoperative days; *p* < 0.001), and shorter duration of hospitalization (LCR 12.0 days vs. OC 13.7 days; *p* < 0.001). The frequency of surgical site infection was lower with LCR (2.1%) than with open surgery (7.4%). However, those studies excluded patients over 90 years old. In our study, the LCR group showed the same good short-term results seen in previous RCTs, suggesting that LCR is a useful approach for patients over 90 years old.

The frequency of postoperative complications among colorectal cancer patients over 80 years old has been reported to range from 21 to 46.4% [15–17]. The present study of patients ≥90 years old also showed a high value of 40.2%. In general, the tissues in elderly individuals are fragile and weak, and blood supply and tissue healing are known to worsen with age. Past reports have indicated older age as a risk factor for infectious complications in a variety of diseases [18–20]. Postoperative infectious complications lead to longer duration of hospitalization, higher medical costs, and, in the elderly, reduced activities of daily living [21]. In this study, among very elderly patients at high risk of infectious complications, the LCR group showed a significantly lower frequency of infectious complications, particularly pneumonia and urinary tract infections. Prior reports have shown that laparoscopic surgery can reduce wound pain and the need for postoperative analgesia [11, 21]. The small wound from laparoscopic surgery, which reduces wound pain, may enable early weaning and early removal of urinary catheters and other devices, which may in turn contribute to reducing the frequency of pneumonia and urinary tract infections. In fact, duration of hospitalization was significantly shorter in the group of patients who did not develop infectious complications in this study.

With regard to laparoscopic surgery for the elderly, some reports have described the effects of laparoscopic insufflation pressure on cardiopulmonary function and the risk of increased postoperative complications [22]. Mortality among elderly colorectal cancer patients has been reported as 1.1–15.6% [15, 16, 23, 24]. On the other hand, recent reports have shown that despite the fact that elderly patients have a higher frequency of cardiovascular disease as a comorbidity and higher risk of anesthesia, short-term outcomes and oncologic treatment efficacy have been reported as comparable to those of non-elderly patients [25, 26].

In the present study, no intraoperative problems or postoperative cardiopulmonary complications were encountered in the LCR group. No postoperative cardiopulmonary complications or in-hospital deaths occurred. One possible explanation is that this study was performed on a relatively recent patient group, and improvements in modern anesthesia techniques and perioperative management now allow even very elderly patients to be treated relatively safely.

The strengths of this study were that it was a multicenter study using real-world data based on actual clinical practice and that the analysis was based on relatively recent data, from a time when LCR had become widespread. Several limitations to the present study need to be kept in mind. First, this was a retrospective study of a small number of patients, so some selection biases could have still been present. Second, because of the small number of patients in this study, covariate adjustment between the LCR and OP groups was insufficient, and patient backgrounds were not well matched. A larger study is needed in the future. Third, the indications for laparoscopic surgery depended on the choices of the surgeon according to the general condition of the patient or progression of cancer.

## Conclusions

Despite the limitations of the study, LCR appears useful for patients ≥90 years old as a procedure that reduces the risk of postoperative infectious complications.

## Data Availability

The datasets used and/or analyzed during the current study are available from the corresponding author on reasonable request.

## References

[1] Chan TY, Foo CC, Law WL, Lo O (2019). Outcomes of Colorectal cancer Surgery in the nonagenarians: 20-year result from a tertiary center. BMC Surg.

[2] Sung H, Ferlay J, Siegel RL, Laversanne M, Soerjomataram I, Jemal A (2021). Global Cancer statistics 2020: GLOBOCAN estimates of incidence and Mortality Worldwide for 36 cancers in 185 countries. CA Cancer J Clin.

[3] Bray F, Ferlay J, Soerjomataram I, Siegel RL, Torre LA, Jemal A (2018). Global cancer statistics 2018: GLOBOCAN estimates of incidence and mortality worldwide for 36 cancers in 185 countries. CA Cancer J Clin.

[4] Alves A, Panis Y, Mathieu P, Mantion G, Kwiatkowski F, Slim K (2005). Postoperative mortality and morbidity in French patients undergoing colorectal Surgery: results of a prospective multicenter study. Arch Surg.

[5] Turrentine FE, Wang H, Simpson VB, Jones RS (2006). Surgical risk factors, morbidity, and mortality in elderly patients. J Am Coll Surg.

[6] Lacy AM, García-Valdecasas JC, Delgado S, Castells A, Taurá P, Piqué JM (2002). Laparoscopy-assisted colectomy versus open colectomy for treatment of non-metastatic colon Cancer: a randomised trial. Lancet.

[7] Nelson H, Sargent DJ, Wieand HS, Fleshman J, Anvari M, Stryker SJ (2004). A comparison of laparoscopically assisted and open colectomy for colon Cancer. N Engl J Med.

[8] Green BL, Marshall HC, Collinson F, Quirke P, Guillou P, Jayne DG (2013). Long-term follow-up of the Medical Research Council CLASICC trial of conventional versus laparoscopically assisted resection in Colorectal cancer. Br J Surg.

[9] Buunen M, Veldkamp R, Hop WC, Kuhry E, Jeekel J, Haglind E (2009). Survival after laparoscopic Surgery versus open Surgery for colon Cancer: long-term outcome of a randomised clinical trial. Lancet Oncol.

[10] Hewett PJ, Allardyce RA, Bagshaw PF, Frampton CM, Frizelle FA, Rieger NA (2008). Short-term outcomes of the Australasian randomized clinical study comparing laparoscopic and conventional open surgical treatments for colon Cancer: the ALCCaS trial. Ann Surg.

[11] Yamamoto S, Inomata M, Katayama H, Mizusawa J, Etoh T, Konishi F (2014). Short-term surgical outcomes from a randomized controlled trial to evaluate laparoscopic and open D3 dissection for stage II/III colon Cancer: Japan Clinical Oncology Group Study JCOG 0404. Ann Surg.

[12] Ghanem M, Garthmann J, Redecker A, Ahrberg-Spiegl AB, Fakler JKM, Spiegl UJA (2021). Management of pertrochanteric fractures in patients over 90 years: In-hospital mortality rate, Complications and associated risk factors. BMC Musculoskelet Disord.

[13] Issa M, Kiening KL, Unterberg AW, Scherer M, Younsi A, Fedorko S (2021). Morbidity and mortality in patients over 90 years of Age following posterior stabilization for Acute traumatic odontoid type II fractures: a retrospective study with a Mean Follow-Up of three years. J Clin Med.

[14] Hashimoto S, Tominaga T, Nonaka T, Hamada K, Araki M, Takeshita H (2020). The C-reactive protein to albumin ratio predicts postoperative Complications in oldest-old patients with Colorectal cancer. Int J Colorectal Dis.

[15] Tokuoka M, Ide Y, Takeda M, Hirose H, Hashimoto Y, Matsuyama J (2016). Single-port versus multi-port laparoscopic Surgery for colon Cancer in elderly patients. Oncol Lett.

[16] Monson K, Litvak DA, Bold RJ (2003). Surgery in the aged population: surgical oncology. Arch Surg.

[17] Kim KI, Park KH, Koo KH, Han HS, Kim CH (2013). Comprehensive geriatric assessment can predict postoperative morbidity and mortality in elderly patients undergoing elective Surgery. Arch Gerontol Geriatr.

[18] Fung M, Jacobsen E, Freedman A, Prestes D, Farmakiotis D, Gu X (2019). Increased risk of infectious Complications in older patients with indolent Non-hodgkin Lymphoma exposed to Bendamustine. Clin Infect Dis.

[19] Pham C, Kuten SA, Knight RJ, Nguyen DT, Graviss EA, Gaber AO (2020). Assessment of infectious Complications in elderly kidney transplant recipients receiving induction with anti-thymocyte globulin vs basiliximab. Transpl Infect Dis.

[20] Ittisanyakorn M, Ruchichanantakul S, Vanichkulbodee A, Sri-On J (2019). Prevalence and factors associated with one-year mortality of infectious Diseases among elderly emergency department patients in a middle-income country. BMC Infect Dis.

[21] Tominaga T, Takeshita H, Arai J, Takagi K, Kunizaki M, To K (2015). Short-term outcomes of laparoscopic Surgery for Colorectal cancer in oldest-old patients. Dig Surg.

[22] Uemura N, Nomura M, Inoue S, Endo J, Kishi S, Saito K (2002). Changes in hemodynamics and autonomic nervous activity in patients undergoing laparoscopic cholecystectomy: differences between the pneumoperitoneum and abdominal wall-lifting method. Endoscopy.

[23] Verweij NM, Schiphorst AH, Maas HA, Zimmerman DD, van den Bos F, Pronk A (2016). Colorectal Cancer resections in the Oldest Old between 2011 and 2012 in the Netherlands. Ann Surg Oncol.

[24] Nakanishi R, Oki E, Sasaki S, Hirose K, Jogo T, Edahiro K (2018). Sarcopenia is an Independent predictor of Complications after Colorectal cancer Surgery. Surg Today.

[25] Peltrini R, Imperatore N, Carannante F, Cuccurullo D, Capoulupo GT, Bracale U (2021). Age and comorbidities do not affect short–term outcomes after laparoscopic rectal cancer resection in elderly patients. A multi–institutional cohort study in 287 patients. Updates Surg.

[26] Roscio F, Boni L, Clerici F, Frattini P, Cassinotti E, Scandroglio I (2016). Is laparoscopic Surgery really effective for the treatment of colon and rectal cancer in very elderly over 80 years old? A prospective multicentric case–control assessment. Surg Endosc.

